# Semaglutide therapy and iatrogenic thyrotoxicosis

**DOI:** 10.1530/EDM-25-0065

**Published:** 2025-07-10

**Authors:** Maxim John Levy Barnett, Sarah Eidbo, Ana Rivadeneira

**Affiliations:** ^1^ Internal Medicine, Jefferson Einstein Hospital, Philadelphia, Pennsylvania, USA; ^2^ Endocrinology, Jefferson Einstein Hospital, Philadelphia, Pennsylvania, USA

**Keywords:** thyroid, obesity, diabetes

## Abstract

**Summary:**

Levothyroxine is the backbone of hypothyroidism treatment. The dosage of levothyroxine varies; however, as an estimate, an average adult patient will require 1.6 micrograms per kilogram of body weight. We present the case of a patient with hypothyroidism, controlled on a stable dosage of levothyroxine, who subsequently began semaglutide therapy for obesity. She developed rapid weight loss and presented with palpitations as her main symptoms. Both clinical and biochemical analyses demonstrated new hyperthyroidism. With the weight loss, it was deemed that her levothyroxine dosage was no longer appropriate for her new weight and was over-suppressing her thyroid function (iatrogenic hyperthyroidism), requiring a dosage reduction. With follow-up, both clinical assessment and biochemical studies noted a reduction in the suppression of the thyroid axis. This case highlights the importance of considering a dosage reduction of levothyroxine when patients lose significant weight (such as with concurrent obesity medications), to prevent iatrogenic hyperthyroidism.

**Learning points:**

- Weight loss (pharmacological or surgical) can be associated with a reduction in TSH; it is unclear whether this is directly related to the reduction in body mass index.
- Hypothyroid patients on levothyroxine who are treated for obesity should be monitored for clinical and biochemical evidence of hyperthyroidism, and clinicians should anticipate that a dosage reduction may be required.
- The mechanism leading to iatrogenic hyperthyroidism in hypothyroid patients with weight loss therapy is unknown but believed to occur either from increased absorption of the medication or as a result of the weight loss itself (posing a supratherapeutic level of levothyroxine).

## Background

Hypothyroidism is defined as the state of an underactive thyroid gland; while a myriad of causes are known, the most common cause within the United States is autoimmune thyroiditis (Hashimoto’s thyroiditis), first described in 1912 by Japanese physician Hakaru Hashimoto (1). Management involves replacement of thyroid hormone, often achieved by oral levothyroxine (synthetic thyroxine). Current data suggest that around seven percent of the American population currently have a prescription for daily levothyroxine (accounting for nearly 23 million Americans) (2). Guidelines recommend an individualized approach to initiating levothyroxine based on patient characteristics, such as age and comorbidities; for example, the elderly and those with coronary artery disease are advised to begin at a lower dose. In the absence of contraindications, a recommended 1.6 micrograms per kilogram body weight is advised as the required dosage of levothyroxine for replacement therapy (3). The efficacy of thyroid replacement is monitored through measurement of thyroid-stimulating hormone (TSH) in cases of primary hypothyroidism (and by monitoring serum free T4 in cases of secondary/tertiary hypothyroidism) (4). Causes of persistent elevation in TSH suggest inadequate control and are most commonly because of nonadherence, or malabsorption of thyroxine. Obesity is often associated with elevated TSH levels, and such patients often require a higher dosage of levothyroxine to control the underlying hypothyroidism (5). When weight loss is achieved, through medical or surgical interventions, it has been demonstrated that TSH levels often decline. As a result, when patients who are obese with hypothyroidism (on levothyroxine supplementation) lose weight, a dosage reduction should be anticipated. We present the case of a patient with hypothyroidism on levothyroxine with iatrogenic hyperthyroidism after commencing semaglutide and a resultant rapid reduction in weight.

## Case presentation

A female patient in her 50s presented to the endocrine clinic to discuss therapeutic options for weight loss. She had a past medical history of type 2 diabetes mellitus, hypothyroidism, and obesity. The duration of hypothyroidism was unknown; however, she was stable on 125 micrograms of levothyroxine, with her most recent thyroid hormone profile demonstrating a TSH of 1.02 mIU/L (reference range: 0.45–4.5 mIU/L) and a free T4 of 1.53 ng/dL (reference range: 0.82–1.77 ng/dL). Her only other medication at the time was metformin 500 milligrams, which was administered once daily. At the office visit, her glycemic control was noted to have worsened (HbA1c 7.3% from 6.9% previously). Her weight at the office was 223 pounds (101.2 kilograms), above her ideal body weight. After shared decision-making, she agreed to increase her metformin to 500 milligrams twice daily and commence semaglutide, starting at 0.25 milligrams weekly for 1 month and subsequently increasing to 0.5 milligrams.

She was seen as a follow-up in the clinic 5 months later. At the follow-up appointment, she was noted to have lost 39 pounds (17.7 kilograms) (current weight 184 pounds/83.5 kilograms) and had demonstrated a significant improvement in her glycemic control (HbA1c 5.8%). While pleased with her progress, a review of systems identified symptoms of palpitations, perspiration, and heat intolerance. A physical examination was largely unremarkable apart from regular sinus tachycardia.

## Investigation

The primary concern was for over-suppression of the hypothalamic–pituitary–thyroid axis as a result of the excessive weight loss. Thyroid function tests were obtained, demonstrating an unmeasurable TSH (<0.005 mIU/L; reference range: 0.45–4.5 mIU/L), consistent with iatrogenic hyperthyroidism.

## Treatment

The patient was advised to reduce her levothyroxine to 100 micrograms daily. Furthermore, as she was tolerating the semaglutide without adverse events, the dosage was increased to 1 milligram weekly.

## Outcome and follow-up

**Figure 1:**
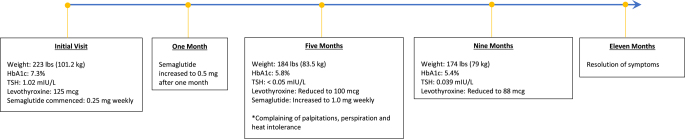
Timeline of events. kg, kilograms; lbs, pounds; mcg, micrograms; mg, milligram; mIU/L, milli-international units per liter.

Following reduction of the levothyroxine dosage to 100 milligrams daily, the patient returned to the clinic 4 months later (9 months after commencement of semaglutide). At this visit, the symptoms of thyrotoxicosis had resolved, and she had demonstrated a further weight loss of ten pounds (4.5 kilograms) (current weight 174 pounds/78.9 kilograms). A repeat thyroid function panel was obtained, demonstrating an improvement in her TSH (0.0391 mIU/L; reference range: 0.45–4.5 mIU/L), and the dosage of levothyroxine was advised to be reduced further to 88 micrograms. At a telemedicine appointment 2 months later, her symptoms had resolved (Fig. 1).

## Discussion

The case presented demonstrates the importance of adjusting the dosage of levothyroxine for body weight. When weight loss is evident, through either pharmacological or surgical means, patients are at risk of untoward iatrogenic thyrotoxicosis, as the dosage of levothyroxine is in excess of their new body mass index. As demonstrated in our case, a 30% dosage reduction was required following initiation of semaglutide to assist with weight loss. Wilcox and Dril pose a similar presentation of a patient in her 40s with postoperative hypothyroidism following total thyroidectomy for papillary thyroid carcinoma; their patient was maintained on 200 micrograms of levothyroxine daily and was started on semaglutide (titrated to 1 milligram) (6). Over the next 13 months, she was found to be hyperthyroid, requiring frequent dosage adjustments to a final dosage of 150 micrograms after 22 months to achieve a normal TSH, translating to a 25% dosage reduction (6).

The interplay between weight and TSH is complex, with numerous differing hypotheses. In euthyroid individuals, obese patients are often noted to have an elevated TSH; while instinctively one may assume the high TSH to be responsible for the weight gain, it is rather the effect of obesity itself leading to an elevation in TSH (5). The underlying mechanism regarding our case and that of Wilcox and Dril has not been entirely identified; however, two prominent theories include: i) a direct effect upon enhanced absorption of levothyroxine from delayed gastric emptying and ii) a result of weight loss, leading to a supratherapeutic levothyroxine dosage for that body weight (6). Others suggest glucagon-like peptide-1 (GLP-1) receptor agonists have a direct effect upon central inhibition of the hypothalamic–pituitary–thyroid axis, while it has been noted that such receptors are present in the paraventricular nucleus of the hypothalamus (whereby thyrotropin-releasing hormones are present), this theory is less plausible, as one would expect concurrent reductions in free T4, which is not evident in most studies (7). Furthermore, while receptors for GLP-1 are noted within the thyroid gland, they appear to only be expressed within the parafollicular (C) cells and not the follicular cells; moreover, inhibition at the level of the thyroid gland itself would once again demonstrate a reduction in free T4, which has not been noted (7).

In animal studies, Malendowics & Nowak demonstrate a notable reduction in TSH secretion in rats exposed to exenatide (8). In human studies, Kaylee *et al.* assessed 17 patients with hypothyroidism who were exposed to tirzepatide, and at 6 weeks post-exposure, 5/17 patients had suppressed TSH levels, while the remaining 11 patients had reduced (but not suppressed) levels (9). The authors provided a recommendation to monitor TSH levels in patients who are started on tirzepatide to prevent iatrogenic hyperthyroidism as a result (9). Regarding oral semaglutide, Hauge and colleagues analyzed the effect of levothyroxine coadministration, noting a 33% increase in the area under the curve of levothyroxine, suggesting enhanced absorption. One caveat, however, is that patients received supratherapeutic levothyroxine (600 micrograms), limiting overall interpretability (10).

In euthyroid subjects, Köseoglu and colleagues assessed 39 patients with obesity and type 2 diabetes mellitus exposed to exenatide, noting a statistically significant reduction in TSH (alongside thyroid volume); of note, the authors state there was no significant relationship between body mass index reduction and TSH reduction (11). Tee and colleagues performed a study of 112 obese patients with type 2 diabetes mellitus (without a history of thyroid disease) treated with exenatide, noting a significant mean reduction in TSH alongside weight loss; uniquely, the authors state that the patients who did not lose weight did not demonstrate changes in TSH levels (12). Similarly, Sencar *et al.* studied 33 euthyroid patients with diabetes managed with exenatide, noting a statistically significant reduction in TSH without significant changes in free T4, T3, or thyroid volume; again, the authors note no correlation between TSH and body mass index, irrespective of weight loss (13). Contrary to these findings, however, Danciulescu *et al.* failed to identify any significant changes in TSH among 33 euthyroid type 2 diabetes mellitus patients managed with exenatide (14). When studying liraglutide in patients with type 2 diabetes mellitus without thyroid disease, Ye *et al.* demonstrated lower TSH levels with exposure (15).

When considering non-GLP-1 medications, Sari and colleagues assessed sibutramine and orlistat, demonstrating significant reductions in TSH concentrations in obese women (but note, TSH only decreased in obese women who lost more than 10% of their body weight) (16). Numerous studies have further explored metformin, providing mixed results with respect to TSH. In an early trial, Capelli *et al.* demonstrated a significant decrease in TSH with short-term metformin therapy in hypothyroid patients (and those with subclinical hypothyroidism) with type 2 diabetes mellitus, regardless of whether they received levothyroxine therapy (17). The authors subsequently performed another study, analyzing 393 euthyroid patients with type 2 diabetes mellitus (18). Uniquely, patients were stratified to receive no treatment, monotherapy with metformin, or dual therapy with metformin and levothyroxine; the authors noted TSH decreased independently from the basal level in patients on dual therapy, but for those solely exposed to metformin, TSH only decreased if there was a significantly high basal TSH (18).

Apart from medications, a similar phenomenon is noted post-bariatric surgery (albeit again, results are mixed). In the study by MacCuish and colleagues, 55 euthyroid patients failed to demonstrate a significant change in TSH following Roux-en-Y gastric bypass (19). Similarly, Dall’Asta *et al.* assessed 311 euthyroid patients following laparoscopic gastric banding, with no significant changes in TSH noted; both studies, however, did demonstrate a significant increase in free T4 (20). Contrary to these aforementioned studies, Tian and colleagues demonstrated a significant correlation between weight loss and TSH following bariatric surgery (21). While Moulin de Moraes *et al.* noted a significant decrease in TSH post-bariatric surgery, their findings indicate that reductions in TSH are independent of body mass index (22). Zendel and colleagues studied hypothyroid patients retrospectively following bariatric surgery, noting significant decreases in TSH, directly correlating to baseline levels (23). Meta-analyses performed by Azran *et al.*, Najjari *et al.*, and Guan *et al.* demonstrated significant reductions in TSH following bariatric surgery (24, 25, 26).

The intricate relationship between TSH and body weight has not been fully identified. Within the literature, it is evident that a decrease in weight can be associated with a reduction in TSH levels. Whether surgical or pharmacological means are utilized for inducing weight loss, one must anticipate a reduction in TSH levels. In hypothyroid patients, particularly those managed with levothyroxine, there is a risk of iatrogenic hyperthyroidism following excess weight loss, for which a dosage reduction will be required to prevent harm. Further studies are required to identify the exact mechanism pertaining to the development of hyperthyroidism, as in our case report; however, all clinicians should be aware of this potential complication.

## Declaration of interest

The authors declare that there is no conflict of interest that could be perceived as prejudicing the impartiality of the work reported.

## Funding

This research did not receive any specific grant from any funding agency in the public, commercial, or not-for-profit sector.

## Patient consent

Written informed consent for publication of their clinical details was obtained from the patient via consent form.

## Author contribution statement

M Barnett, S Eidbo, and A Rivadeneira were involved in the care of the patient. M Barnett and S Eidbo wrote the manuscript and reviewed the literature. The draft was finalized and revised by M Barnett and A Rivadeneira.
